# Detroit Gynæcological Society

**Published:** 1886-09-20

**Authors:** 


### DETROIT GYNÆCOLOGIGAL SOCIETY.

The society met at the office of Dr. Andrews, the president occupying the chair, February 3, 1886.

### WRITTEN COMMUNICATIONS.

Dr. Jennings presented a paper on "Anæsthetics in Labor," which advocated the proper use of chloroform in all cases of painful parturition.

### DISCUSSION.

Dr. Hutton said that he could endorse the statement made by the reader. He had never seen bad results from the use of chloroform. Occasionally a patient objects to the use of an anæsthetic, but usually they are desirous of the relief which chloroform affords. He frequently allows the patient to administer it to herself, inhaling a few drops at a time as she feels the pain advancing.

Dr. Warner: I do not think a routine practice good in any condition. I do not use chloroform in all cases, for I think there are a greater proportion of physiological labors than Dr. Jennings seems to have met with. I think I have seen a good many cases where there has been a pretty sharp gush of blood, necesitating the application of ice, etc., to check it, which I thought were due to the chloroform administered. It is often stated that ether in labor is useless unless it is pushed to full anæsthesia. I do not think that anyone who has had experience with ether will agree with this. I have seen great relief from pain when the patient has been only partially under its effect.

There are many conditions in which I prefer ether to chloroform; one of these is stricture of the internal os, on which it seems to me to have a good effect in relaxing.

Dr. Jenks: The subject which the paper brings before us is a very important one, and suggests many things worthy of our consideration. As to the kind of anæsthetic, I have no hesitation in stating that there is none which should be used in child-birth but chloroform. I condemn the A. C. E. mixture unhesitatingly.

The reason I would not give ether is because of its pernicious effects upon the mother. It must be given to complete anæsthesia.

Its effect upon the child is also bad. I have seen two children on the same street, born on the same day, both deeply asphyxiated. The death of the child in each instance was produced by the ether given its mother.

In one such case the nurse had given the ether, and the child was born asphyxiated, and although vigorous efforts were made at resuscitation, it remained for twelve or more hours partially anæsthetized. Afterward its color changed, the condition disappeared, and the child grew up.

We must draw the line between surgical and obstetrical anæsthesia.

My practice has been not to give an anæsthetic during the first stage of labor. During the second stage I give chloroform mixed with cologne or alcohol, if either is at hand. Later on, if necessary, the stronger chloroform may be given. If the pure chloroform is given at first, there is no more peace unless it is kept up. It is much the better to begin with the diluted solution. In the first stage of labor chloral hydrate is often of service. When operative procedure is undertaken, the administration should be pushed to surgical anæsthesia.

The one thing that I fear is post partum hæmorrhage; and that chloroform does have the effect I can personally testify.

The effect of ergot is not quick enough to overcome this tendency.

Dr. Longyear: I am in favor of anæsthetics in labor, and decidedly prefer chloroform to any other, perhaps because it is quick to act. I administer it in the majority of my cases. In a few cases which I have had I have noticed post partum hæmorrhage, which I ascribed to the chloroform.

In the case of a slight, fragile woman weighing eighty-five pounds, chloroform was administered after the head had passed os. Immediately after labor the womb became hard and all seemed well. I held my hand over the womb, and it seemed to relax and contract with great regularity. I continued this for one-half hour, as is my custom, and then put on the binder. While I was still in the room, the patient complained of feeling dizzy. I examined and found the womb was at the umbilicus, and I was able to squeeze out some blood and clots. In spite of pressure the hemorrhage continued until I applied electricity (Faradic current).

I can only ascribe this hemorrhage to the chloroform, but it might have been due to other causes.

Dr. Carstens said that he was not in the habit of using chloroform in all cases. He thought other anodynes good; a dose of opium or chloral hydrate being often of great service. Chloroform is undoubtedly the anæsthetic to be employed. He considered the A. C. E. mixture a dangerous combination. Chloroform, no doubt, checks the pains. When it is administered in order to rectify a malposition, and then removed, he found that the pains did not come up again, and a resort to instrumental delivery is often necessary.

Some women will not take an anæsthetic, even if forceps have to be applied. In these cases he thought opium of service.

He did not feel quite sure that opium produces flooding; but in case hemorrhage occurs, ergot, except hypodermically, is not to be relied on.

Dr. Chittick had given little attention to the subject, but it occurred to him that if chloroform relaxed the other muscles, why would it not those of the uterus? In stricture of the os, he preferred digital dilatation, or vaginal douches of hot water.

Dr. Manton remarked that he had used both ether and chloroform, and had seen the latter given quite extensively abroad, especially in operative cases. He did not, however, recall a single case of post partum hemorrhage from its use, although a tendency to this might reasonably be supposed to exist when the anæsthesia was profound, and the smooth fibres of the uterus became involved in its action.

But one death from chloroform administered in childbirth had come to his notice. In this case, which occurred in a Western city, the anæsthetic had been intrusted by the physician to the nurse, while he himself remained in another part of the house.

The action of chloroform upon the child must not be forgotten. Zweifel and others have detected chloroform in the fœtal blood, and Schatz has seen several children born deeply asphyxiated, in cases where chloroform has been given.

An anæsthetic is undoubtedly superfluous in a great many instances, and, therefore., routine practice in the administration of such should be avoided.

Dr. Andrews: The paper which we have heard presents the rose-colored side of chloroform in labor. My own experience dates back to the time before chloroform was used as promiscuously as at present; when not quite so much dependence was placed upon it as now. What has been already said in regard to routine is most important. Each case should be studied by itself. In some women an anæsthetic produces a cessation, or diminished force of contraction, without regard to stage of labor. Working women do not seem to suffer the pangs of labor so much as women of a higher class, with a more delicate organization. In the case of the former it is often an absurd refinement to give an anæsthetic.

In my experience, chloroform seems to predispose to hemorrhage. I can recall four cases in which it seems to me the involuntary muscular fibres of the uterus were relaxed, resulting in flooding.

Of morphine, opium, and other anodynes I think highly. When delay occurs, when there is a rigid os, or the like, they often act very well.

I do not find it necessary to use chloroform in all forceps cases. In many instances the nausea and effects of the anæsthetic are more disagreeable than the pain produced by forceps. I have performed podalic version with and without anæsthetic, so that I do not think it always necessary in this condition.

Dr. Jennings: I remember a few cases in my own practice where I have thought that a tendency to hemorrhage was manifested after chloroform had been given. In cases where I have applied forceps the uterus has contracted well in every instance. This has served to strengthen my confidence in chloroform. I did not intend to advocate the indiscriminate use of anæsthetics in labor.

The conditions requiring the use of chloroform are mostly found in primiparæ, in whom the second stage is nearly always prolonged.—*American Lancet.*

